# Evidence for Positive Selection on the Leptin Gene in Cetacea and Pinnipedia

**DOI:** 10.1371/journal.pone.0026579

**Published:** 2011-10-27

**Authors:** Li Yu, Wei Jin, Xin Zhang, Ding Wang, Jin-song Zheng, Guang Yang, Shi-xia Xu, Soochin Cho, Ya-ping Zhang

**Affiliations:** 1 Laboratory for Conservation and Utilization of Bio-resource and Key Laboratory for Microbial Resources of the Ministry of Education, Yunnan University, Kunming, China; 2 Institute of Hydrobiology, Chinese Academy of Sciences, Wuhan, China; 3 Jiangsu Key Laboratory for Biodiversity and Biotechnology and Institute of Genetic Resources, College of Life Sciences, Nanjing Normal University, Nanjing, China; 4 Department of Biology, Creighton University, Omaha, Nebraska, United States of America; 5 State Key Laboratory of Genetic Resources and Evolution, Kunming Institute of Zoology, Chinese Academy of Sciences, Kunming, China; University of Arkanas, United States of America

## Abstract

The *leptin* gene has received intensive attention and scientific investigation for its importance in energy homeostasis and reproductive regulation in mammals. Furthermore, study of the *leptin* gene is of crucial importance for public health, particularly for its role in obesity, as well as for other numerous physiological roles that it plays in mammals. In the present work, we report the identification of novel *leptin* genes in 4 species of Cetacea, and a comparison with 55 publicly available *leptin* sequences from mammalian genome assemblies and previous studies. Our study provides evidence for positive selection in the suborder Odontoceti (toothed whales) of the Cetacea and the family Phocidae (earless seals) of the Pinnipedia. We also detected positive selection in several leptin gene residues in these two lineages. To test whether leptin and its receptor evolved in a coordinated manner, we analyzed 24 leptin receptor gene (*LPR*) sequences from available mammalian genome assemblies and other published data. Unlike the case of leptin, our analyses did not find evidence of positive selection for *LPR* across the Cetacea and Pinnipedia lineages. In line with this, positively selected sites identified in the *leptin* genes of these two lineages were located outside of leptin receptor binding sites, which at least partially explains why co-evolution of leptin and its receptor was not observed in the present study. Our study provides interesting insights into current understanding of the evolution of mammalian *leptin* genes in response to selective pressures from life in an aquatic environment, and leads to a hypothesis that new tissue specificity or novel physiologic functions of *leptin* genes may have arisen in both odontocetes and phocids. Additional data from other species encompassing varying life histories and functional tests of the adaptive role of the amino acid changes identified in this study will help determine the factors that promote the adaptive evolution of the *leptin* genes in marine mammals.

## Introduction

Leptin is an adipose tissue-derived circulating hormone with multiple functions pivotal in regulating energy homeostasis and reproductive functions in mammals [1], [2], [3], [4], [5], [6], [7]. Increases in *leptin* signal levels act on the central nervous system to inhibit food intake and/or regulate energy expenditure to maintain constancy in adipose mass [1], [4]. Mutations in the *leptin* gene are associated with a myriad of hormonal and metabolic alterations [1], [8]. In humans and mice, leptin deficiency has been shown to cause obesity and diabetes [1], [9], [10], [11], [12].

Evidence of rapid sequence evolution in *leptin* genes has been documented in seals [13], plateau pikas [14], [15] and primates [16], [17], [18], [19], [20], [21], [22], [23], [24]. It has been hypothesized that leptin function in these mammals may have undergone modification [13], [22]. For example, Siltberg and Liberles (2002) proposed that positive selection in primate leptin may be driven by dietary and/or reproductive changes during the evolution of primates relative to other mammals [22]. Therefore, the study of leptin genes in mammals encompassing different evolutionary histories will contribute to our understanding of other physiological roles of leptin.

Marine mammals are a diverse group of 120 species including cetaceans (whales, dolphins, and porpoises), sirenians (manatees and dugong), and pinnipeds (true seals, sea lions and walrus) [25]. Compared with their terrestrial counterparts, marine mammals are remarkable and evolutionarily significant in terms of their adaptation to an aquatic environment, yet there have been few studies of the genetic changes underlying adaptation to an aquatic lifestyle [26], [27], [28]. Interestingly, a recent study by Hammond et al. (2005) revealed a substantially larger number of amino acid substitutions in seal leptin sequences than in other mammals, and also demonstrated unusual expression of leptin in the seal lung [13]. They proposed a role for leptin in seal respiratory physiology in addition to its common role in energy balance as in other mammals. These observations raised important questions about (1) whether excessive amino acid substitutions in seal leptin were due to positive selection and (2) our lack of information about the evolutionary history of leptin genes in other marine mammalian lineages.

Therefore, we sequenced *leptin* genes from four cetacean species representing three families of the suborder Odontoceti (toothed whales) and one family of the suborder Mysticeti (baleen whales). In addition, 55 publicly available *leptin* sequences from mammalian genome assemblies and previous studies, including 3 pinniped species from the families Otariidae (sea lions) and Phocidae (seals), were included in our analyses to address the issue. Since leptin exerts its physiological effects on energy balance via direct binding to the leptin receptor (LPR) [2], [10], [29], we analyzed publicly available 24 *LPR* gene sequences to investigate whether leptin and its have receptor co-evolved.

## Results

### Leptin and LPR Gene Sequences

We obtained the complete coding sequences of *leptin* genes (501 bp) from all four cetaceans (See the Materials and Methods for the species list). They all show features typical of leptin, including an amino-terminal signal peptide of 21 amino acids and a mature protein of 146 amino acids, with four α-helices (helices A–D) and a distorted helix E in the CD loop. The amino acid sequences of the mature peptides encoded by these genes are shown in Figure S1.

In addition to these newly determined cetacean *leptin* genes, the amino acid sequences of the *leptin* and *LPR* genes that are obtained from database searches also have characteristic features of leptin (as described above; Figure S1) and the LPR (Figure S2). The LPR coding sequences span 3480–3501 bp, and include codons for the signal peptide (66 bp) and the mature peptide (3414–3435 bp) consisting of extracellular, transmembrane, and intracellular regions (Figure S2).

### Phylogenetic Analysis

Neighbor-Joining (NJ) trees of the *leptin* and *LPR* genes are shown in Figure S3 and Figure S4, respectively. Maximum-parsimony and Bayesian analyses yielded similar tree topologies and levels of nodal support (See Figure S3 and Figure S4). Both *leptin* and *LPR* gene trees supported the monophyly of each of the eight placental mammalian orders included here, but they did not resolve the relationships among these orders, possibly due to the small number of nucleotide sites used.

### Positive Selection in Leptin Genes of Cetacea and Pinnipedia

**Figure 1:**
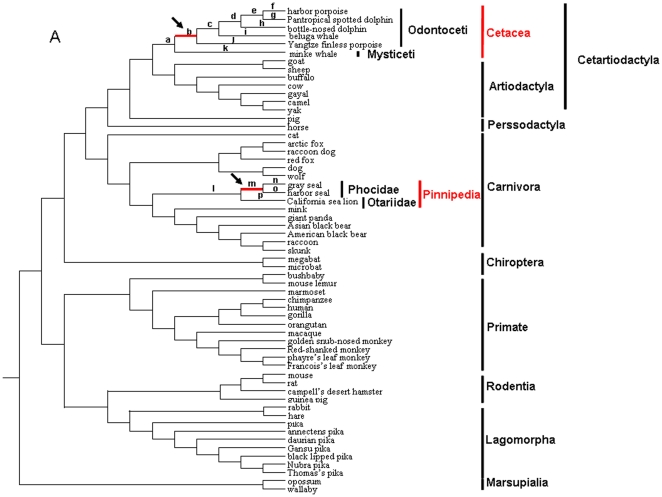
Phylogenetic trees of *leptin* gene used for codon-based maximum likelihood analysis in PAML. Tree topology corresponds to those from Murphy et al. (2001a) and Vogel (2005). Use of the alternative trees (Murphy et al. 2001b, 2007; Springer et al. 2004; Beck et al. 2006) gave essentially the same results. Branches a–p in the tree are used in the branch-site models tests. The thick and red branches (branches b and m) are those with significant evidence of positive selection in PAML analyses.

From the leptin gene tree shown in Figure S3, we noticed a long branch leading to the Phocidae family of Pinnipedia, suggesting rapid rates of sequence evolution. This result is consistent with that of Hammond et al. (2005) where a substantially large number of amino acid substitutions in seal leptin sequences were observed [13]. We performed codon-based maximum likelihood analyses for the leptin genes of Cetacea and Pinnipedia (Tables 1 and S2) to determine whether rapid evolution in Phocidae leptin was driven by positive selection, and to examine the selective pattern of Cetacean leptins. Interestingly, the LRT tests based on the branch-site models for all of the branches in Cetacea and Pinnipedia (16 branches in total, a-p as indicated in Figure 1) suggest there was significant evidence of positive selection along both the lineage leading to the common ancestor of the four Odontoceti species in Cetacea (branch b; 2ΔL = 11.046212, p = 0.0009), and that leading to the common ancestor of the two Phocidae species in Pinnipedia (branch m; 2ΔL = 11.165508, p = 0.0008) (Table 1). After performing Bonferroni correction for multiple testing, the LRT tests were still significant in both branches (p<0.003). Therefore, our analyses suggest that positive selection has operated on *leptin* genes in both Cetacea and Pinnipedia during mammalian evolution.

**Table 1:** PAML analyses for *leptin* gene and evidence of positive selection for Odontoceti and Phocidae.

Models	*lnL* ^a^	Parameter Estimates	2Δ*L* ^b^(P value)	Positively Selected Sites
**Branch-site models (test 2)^c^**				
**Branch b (ancestral Odontoceti)**				
Null	−2743.730055	*ω*_0_ = 0.17773 *ω*_1_ = 1 *ω*_2_ = 1 p_0_ = 0.29440 p_1_ = 0.10628 p_2a_ = 0.44034 p_2b_ = 0.15897		
Alternative	−2738.206949	*ω*_0_ = 0.17871 *ω*_1_ = 1 ***ω***_**2**_ ** = 999.00000** p_0_ = 0.72841 p_1_ = 0.25653 p2a = 0.01114, p2b = 0.00392	**11.046212 (P = 0.0009)**	**H46T (0.975)**
**Branch m (ancestral Phocidae)**				
Null	−2731.716272	*ω*_0_ = 0.15465 *ω*_1_ = 1 *ω*_2_ = 1 p_0_ = 0 p_1_ = 0 p_2a_ = 0.75775, p_2b_ = 0.24225		
Alternative	−2726.133518	*ω*_0_ = 0.15317 *ω*_1_ = 1 ***ω***_**2**_ ** = 14.56744** p_0_ = 0.43153 p_1_ = 0.14201 p2a = 0.32087, p2b = 0.10559	**11.165508 (P = 0.0008)**	**S31C (0.974); Q34P (0.980);L45V (0.972);L49R (0.979);S50T(0.975);T57I (0.996); N72S (0.977); S77A (0.977); E80A (0.976); D85A (0.971);H88R(0.958);S94A (0.970);L104S (0.995);E115R (0.999)**

a lnL is the log-likelihood scores; b likelihood ratio test (LRT) to detect positive selection; c branch b and m are the lineages leading to ancestral Odontoceti and ancestral Phocidae, respectively, in Figure 1.

As summarized in Table 1, the Bayesian approach in PAML predicted one site, site 46 (corresponding to numbers in leptin alignment of Figure S1), as positively selected for branch b with a high Bayesian posterior probability of 0.975. This site is Threonine (T) in all Odontoceti *leptin* sequences, whereas for Mysticeti *leptin*, it is Leucine (L). For branch m, there are fourteen sites in total identified to be adaptive with posterior probability larger than 0.95. Interestingly, site 46 was also detected to be under positive selection in Phocidae, but with moderate Bayesian posterior probability of 0.886.

Based on these sequence rate and identification of positively selected sites, we conclude that adaptive amino acid substitutions in the leptin gene occurred more frequently in the Phocidae lineage than in the Odontoceti lineage.

### LPR Gene Evolution in Cetacea and Pinnipedia

**Figure 2:**
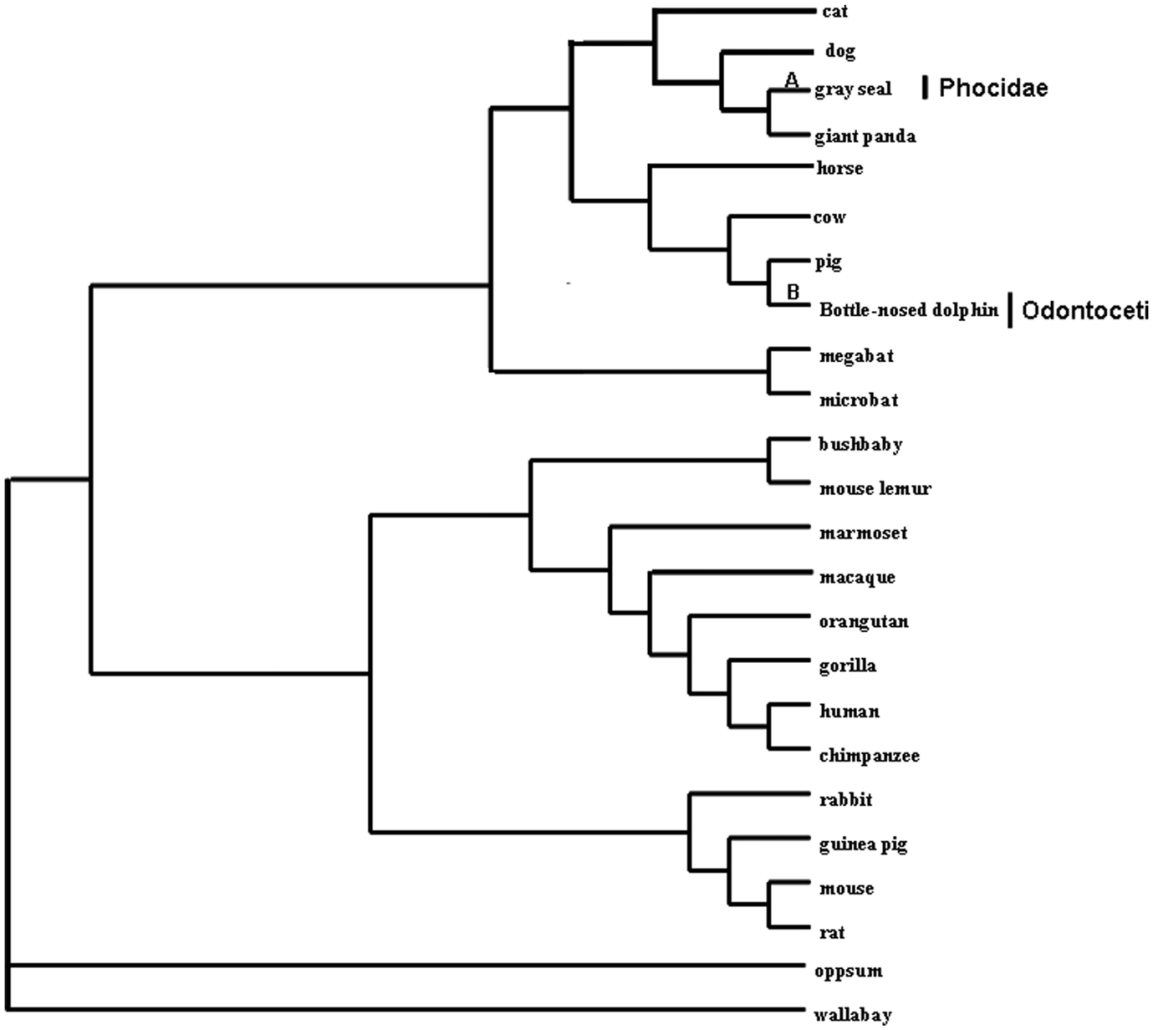
Phylogenetic trees of leptin receptor gene (*LPR*) used for codon-based maximum likelihood analysis in PAML. Tree topology corresponds to those from Murphy et al. (2001a) and Vogel (2005). Use of the alternative trees (Murphy et al. 2001b, 2007; Springer et al. 2004; Beck et al. 2006) gave essentially the same results. Branches A and B in the tree are used in the branch-site models tests.

Due to the complex multi-exon structure of the *LPR* gene (20 exons in mammalian *LPR* genes), fewer mammalian *LPR* gene sequences were available from the published data than leptin sequences. For Cetacea and Pinnipedia, only the *LPR* gene sequences from *Halichoerus grypus* (gray seal; branch A in Figure 2) and *Tursiops truncatus* (bottle-nosed dolphin; branch B in Figure 2) are included in the two lineages of Phocidae and Odontoceti, respectively. From the *LPR* gene tree shown in Figure S4, we found, in contrast to the leptin gene tree, that there were no significant rate changes for the *LPR* genes of Pinnipedia compared with those of most other mammals.

The same model tests as described in the *leptin* gene analyses were performed for these 24 *LPR* genes. As seen in Table 2, none of the LRT tests for the LPR genes were significant. In addition, we conducted analyses based on only cytokine receptor homology domain 2 (CRH2) located in the extracellular region of the *LPR* genes. Previous structural and mutational studies have shown that the CRH2 domain is the main high-affinity binding site for leptin [30], [31], [32], [33], [34], [35]. However, the LRT tests for the CRH2 domain were not significant either (Table 2). Thus, in contrast to the *leptin* genes, we did not find evidence for positive selection in these cetacean and pinniped *LPR* genes. However, it should be noted that fewer *LPR* genes than *leptin* genes from mammalian species were available in the present analyses. Therefore, future studies with more *LPR* genes, especially those from species represented in the *leptin* dataset but not in the *LPR* dataset here, are necessary to test for positive selection of the *LPR* genes in marine mammals.

**Table 2:** PAML analyses for leptin receptor (*LPR*) gene.

Models	*lnL* ^a^	Parameter Estimates	2Δ*L* ^b^(P value)	Positively Selected Sites
**Branch-site models (test 2)^c^**				
**Dataset 1 (complete mature protein)**				
**Branch A (bottle-nosed dolphin)**				
Null	−24857.51845	*ω*_0_ = 0.15154 *ω*_1_ = 1 *ω*_2_ = 1 p_0_ = 0.65326 p_1_ = 0.25041 p_2a_ = 0.06964, p_2b_ = 0.02669		
Alternative	−24857.32236	*ω*_0_ = 0.15164 *ω*_1_ = 1 *ω*_2_ = 3.91631 p_0_ = 0.70244 p_1_ = 0.26897 p2a = 0.02068, p2b = 0.00792	0.39218 (P = 0.5312)	None
**Branch B (gray seal)**				
Null	−24858.17141	*ω*_0_ = 0.15231 *ω*_1_ = 1 *ω*_2_ = 1 p_0_ = 0.72215 p_1_ = 0.27785 p_2a_ = 0, p_2b_ = 0		
Alternative	−24858.17141	*ω*_0_ = 0.15231 *ω*_1_ = 1 *ω*_2_ = 1 p_0_ = 0.72215 p_1_ = 0.27785 p2a = 0, p2b = 0	0 (P = 1.0000)	None
**Dataset 2 (CRH2 domain)**				
**Branch A (bottle-nosed dolphin)**				
Null	−4027.907504	*ω*_0_ = 0.09390 *ω*_1_ = 1 *ω*_2_ = 1 p_0_ = 0.76166 p_1_ = 0.23834 p_2a_ = 0, p_2b_ = 0		
Alternative	−4027.907504	*ω*_0_ = 0.09390 *ω*_1_ = 1 *ω*_2_ = 1 p_0_ = 0.76166 p_1_ = 0.23834 p_2a_ = 0, p_2b_ = 0	0 (P = 1.0000)	None
**Branch B (gray seal)**				
Null	−4027.844849	*ω*_0_ = 0.09346 *ω*_1_ = 1 *ω*_2_ = 1 p_0_ = 0.72395 p_1_ = 0.22627 p_2a_ = 0.03793, p_2b_ = 0.01185		
Alternative	−4027.844849	*ω*_0_ = 0.15805 *ω*_1_ = 1 *ω*_2_ = 1 p_0_ = 0.72395 p_1_ = 0.22627 p_2a_ = 0.03793, p_2b_ = 0.01185	0 (P = 1.0000)	None

a lnL is the log-likelihood scores; b likelihood ratio test (LRT) to detect positive selection; c branch A and B are the lineages leading to bottle-nosed dolphin and gray seal, respectively, in Figure.

## Discussion

Physiological function and molecular evolution of the leptin gene in marine mammals have been poorly studied. For the first time, we show evidence of positive selection in the *leptin* genes of both Cetacea and Pinnipedia, providing valuable insights for understanding the diverse physiological roles it plays in various mammals and also for how such diversity arose during mammalian evolution. Positive selection on the *leptin* gene detected in Cetacea and Pinnipedia appears to have occurred in ancestral Odontoceti after the Odontoceti-Mysticeti split (about 32 million years ago [36], [37], [38]) and ancestral Phocidae after the Phocidae-Otariidae split (about 23 million years ago [39], [40]), respectively. However, no sign of positive selection was detected in suborder Mysticeti of Cetacea and family Otariidae of Pinnipedia with our limited samples.

In previous physiological assays, leptin in marine mammals has been intensely used as a test case for examining whether leptin plays a significant role in body fat regulation and energy metabolism [41], [42], [43], [44], [45], [46], [47]. Marine mammals provide an interesting study model because they have evolved a fat-based metabolism associated with a blubber layer, the main source of energy stores and essential for body insulation from cold marine waters [48], [49], [50], an adaptation to an aquatic environment and prolonged fasting [43], [51]. All of these studies, however, found no correlation between fat mass and serum leptin levels in seal and sea lion species examined so far. Thus, the primary physiological role of leptin in marine mammals may not be an indicator of body fat and energy reserves. Our study provides indirect evidence in support of this argument: if leptin in marine mammals has contributed to regulation of fat deposits and energy balance, adaptive evolution of leptin should have occurred across all marine mammals, including Odontoceti and Mysticeti (Cetacea), and Phocidae and Otariidae (Pinnipedia). Yet, we reject this hypothesis because this pattern was not seen in our study.

Therefore, the unusual pattern of the *leptin* gene evolution observed in some lineages of Cetacea and Pinnipeds is intriguing, raising the possibility that new tissue specificity or additional physiological functions of the *leptin* genes might have arisen in Odontoceti and Phocidae. A number of previous studies have suggested that leptin is expressed in a variety of tissues and blood fluids besides adipose tissues, including placenta, stomach, bone marrow and mammary epithelium [28], [45], [52], [53], [54], [55], [56], and is involved in growth regulation, gastric function, brain development, hematopoiesis, and inflammation [45], [52], [55], [56], [57]. Hammond et al. (2005) found that the leptin in deep-diving seals was expressed in the lung, and proposed a role of leptin in pulmonary surfactant production related to respiratory physiology. A comparison of the diving behaviors between Odontoceti and Mysticeti in Cetacea [58], [59] reveals that Odontoceti generally seeks larger prey at deeper water depths [60], [61], whereas Mysticeti feed on small organisms near the surface of the water and do not hunt their prey or dive to great depths [62]. Therefore, the observed adaptive evolution of leptin in Odontoceti, but not in Mysticeti, might reflect the deep-diving Odontoceti's increased demand for pulmonary or circulatory adaptations to hypoxic conditions. This hypothesis is consistent with the finding that there is adaptive evolution of leptin in deep-diving Phocidae, but not in shallow-diving Otariidae. In the future, it will be interesting to test the expression pattern of *leptin* in cetacean species and the other marine mammals to determine whether the agent causing the observed positive selection of *leptin* gene in Odontoceti and Phocidae is indeed the adaptive responses to respiratory physiology of deep-diving marine mammals.

In contrast to other cytokines and their receptors, e.g., growth hormone (GH) and the growth hormone receptor (GHR) [63], [64], and prolactin (PRL) and the prolactin receptor (PRLR) [65], the evolutionary dynamics of leptin and its receptor (LPR) remain unknown. It has been shown that some cytokines and their receptors evolve in a coordinated manner [65], which is consistent with the ligand-receptor co-evolution hypothesis. Therefore, one could expect that the *LPR* genes in marine mammals would show adaptive evolution similar to that of the *leptin* gene. However, our study did not detect positive selection in *LPR* gene of Cetacea and Pinnipedia, unlike its ligand, the leptin genes. However, this finding is not entirely unexpected for a reason. Even though leptin exerts its physiological effects on energy balance via direct binding to the leptin receptor (LPR) [2], [10], [28], novel leptin functions other than energy balance in Cetacea and Pinnipedia may be independent of the physical interaction between leptin and its receptor. The discrepancy in evolutionary patterns of *leptin* and *LPR* observed here may have resulted from the multi-functionality of leptin and leptin receptor, supporting the view that the biological roles of leptin vary from species to species [18]. The major novelty of our study is to provide clues about the evolutionary scenarios of leptin/receptor signaling system in marine mammals at the molecular level. In future analyses, the inclusion of more *LPR* sequences from marine mammals and other mammals would make more rigorous tests of the co-evolution of leptin and its receptor genes possible.

In summary, our study of *leptin* not only shows that positive selection has promoted rapid divergence of Phocidae leptin, but also demonstrates adaptive evolution of Odontoceti leptin. These findings increase the current knowledge of evolution and function of leptin in mammals, supporting the possibility that new tissue specificity or more crucial physiologic functions of *leptin* genes may have been developed in both Odontoceti and Phocidae. Furthermore, our study revealed several potentially adaptive amino acid changes, providing a foundation for further experimental investigations (e.g. site-directed mutagenesis) to elucidate functional implications of these substitutions in marine mammals. In the future, evolutionary analyses of *leptin* from a wider range of mammals with varying life histories, including more marine mammals, and functional assays of adaptive amino acid changes, should contribute to better understanding of the ecology and the selective agent(s) acting on the evolution of leptin genes.

## Materials and Methods

### Amplification and Sequencing of Cetacea leptin genes

The *leptin* gene sequences from four cetacean species, i.e., *Delphinapterus leucas* (Beluga whale; family Monodontidae, suborder Odontoceti), *Stenella attenuata* (Pantropical spotted dolphin; family Delphinidae; suborder Odontoceti), *Neophocaena phocaenoides asiaeorientalis* (Yangtze finless porpoise; family Phocoenidae, suborder Odontoceti), and *Balaenoptera acutorostrata* (Minke whale; family Balaenopteridae; suborder Mysticeti), were determined in this study. We obtained the *D. leucas* specimen from Harbin Polarland, which was imported from the Okhotsk Sea in Russia, the *S. attenuata* specimen from Dayawan Bay in Shenzhen, China, the *N. p. asiaeorientalis* specimen from Dongting Lake in Hunan Province of China, and the *B. acutorostrata* specimen from the Zhanjiang Coast in GuangDong Province, China. Among these four specimens, the first was obtained from biopsy, while the other three specimens were obtained from stranded animals. For each sample, total genomic DNA was isolated from blood or frozen tissues using a standard proteinase K, phenol/chloroform extraction method [66].

The *leptin* gene has an approximate length of 4.5 kb, spanning two coding exons and an intron, and was amplified by long PCR using the external primer pair Ceta-F (5′-GACAACCACAAGCAGAAAGCAAATCT-3′ and Ceta-R (5′-GCCTTTGGAA GAAGAGCGGCTTAGAG-3′). PCR amplification was done in a 25-µl reaction mixture containing 5 pM of each primer, 100 µM of each dNTP, 2.5 µl 10ΧLA PCR Buffer, 1.25 units of Takara LA *Taq*® (Takara Biotechnology Co., Ltd), and 100 ng genomic DNA. The PCR amplification reaction was performed with 34 cycles of 10 sec at 97°C, 7 min at 66°C, with an initial step of 1 min at 94°C and a final step of 10 min at 72°C.

Amplified PCR products were purified and sequenced in both directions with an ABI PRISM™ 3700 DNA sequencer (PE Biosystems, USA). The full-length 167- amino-acid coding region consisted of a 21-amino-acid signal peptide and a 146- amino-acid mature peptide obtained by designing additional internal sequencing primers Ceta-R1 (5′-GGGACACCGGACCGTTATG-3′), Ceta-R2 (5′-CGCCCAGG CTCTCCAAGGT-3′), Ceta-F1 (5′-AGTGGAGGGCAGGGTGGTT-3′), and Ceta-F2 (5′-CTTCATCCCTGGGCTCACC-3′). Sequence data were assembled using the Lasergene SeqMan program (DNAStar, Madison, Wisc.) and visually checked for accuracy. The sequences were deposited in GenBank under accession numbers HQ689399-HQ689402.

### Database Searches for leptin and LPR genes

Besides the four cetacean *leptin* sequences, we also collected 55 publicly available *leptin* coding sequences for analysis, including 24 from the EMSEMBLE Genome Database (http://www.ensembl.org/) and 31 from published data (http://www.ncbi. nlm.nih.gov/) (Table S1).

In addition to *leptin* genes, the coding sequences of leptin receptor, *LPR*, genes consisting of a 22- amino-acid signal peptide and a 1140-amino-acid mature peptide were obtained from the same 24 mammalian species with available genome sequences (http://www.ensembl.org/) as those in the *leptin* dataset except for the pika *Ochotona princeps*, whose *LPR* gene is unavailable (Table S1). It has been reported that human and mouse *LPR* have multiple alternatively spliced forms [35], [67], [68], [69], [70], [71], [72], [73], and that the full-length form is essential for leptin signaling [69], [74]. Thus, in those cases where multiple splice variants of *LPR* were available for a species, only the full-length form from that species was used for analysis. In addition, the publicly available *LPR* gene from *Halichoerus grypus* (gray seal) (GenBank Accession No. HM448474) was also included.

### Phylogenetic Reconstructions

Nucleotide sequences from the *leptin* coding region and *LPR* were separately aligned using CLUSTAL X program [75]. A 501-bp alignment of 59 *leptin* sequences and a 3519-bp alignment of 24 *LPR* sequences were used for phylogenetic analyses. The amino acid alignments of their mature peptides are shown in Figure S1 and S2, respectively.

Phylogenetic reconstructions were performed using MEGA4 (Kumar et al. 2008) for neighbor-joining (NJ) analyses, PAUP*4.0b8 [76] for maximum- parsimony (MP) analyses, and MrBayes 3.1.2 [77] for Bayesian inference. In NJ analysis, Kimura's 2-parameter nucleotide model with pairwise deletion option for gaps was used. In MP analysis, a heuristic search strategy was employed with the TBR branch swapping algorithm, random addition of taxa and 1000 replicates per search. The reliability of the tree topologies was evaluated using bootstrap support (BS; [78]) with 1000 replicates for NJ and MP analyses. In Bayesian analyses, the best-fit models of sequence evolution were selected using the Akaike Information Criterion (AIC; [79], [80]) with jModeltest [81]. The chosen models were used in the priors of Bayesian inference. Four Metropolis-coupled Markov chain Monte Carlo (MCMC) analyses were run for 2×10^6^ generations, sampling trees every 100 generations. The average standard deviation of split frequencies was below 0.001 when the run ended. The first 25% were discarded as the burn-in. A 50% majority-rule consensus of post burn-in trees was constructed to summarize posterior probabilities (PP) for each branch. Opossum and wallaby sequences were used as outgroups in all analyses.

### Molecular Evolutionary Analysis

The nonsynonymous to synonymous rate ratio ω (dN/dS) provided an indication of the change of selective pressures. A dN/dS ratio  =  1, <1, and >1 are indicative of neutral evolution, purifying selection, and positive selection at the protein involved, respectively. We applied the codon substitution models implemented in the CODEML program in the PAML package [82]. Only gene regions coding for the mature leptin (438 bp in alignment) and LPR peptide (3453 bp in alignment) were analyzed. All models correct the transition/transversion rate and codon usage biases (F3×4). The ambiguous sites were removed in PAML analysis of *leptin* gene (clean data = yes). Different starting ω values were also used to avoid the local optima on the likelihood surface [83].

The branch-site models accommodating ω ratios to vary both among lineages of interest and amino acid sites were considered here [84], [85]. We used branch-site Model A for stringent testing (test 2) and identification of sites under positive selection along the lineages of interest. Significant differences between models were evaluated by calculating twice the log-likelihood difference following a χ^2^ distribution, with the number of degrees of freedom equal to the difference in the numbers of free parameters between models. The presence of sites with ω>1 is suggested when the positive-selection model (Model A) fits the data significantly better than the corresponding null model (M1a). We used the conservative Bayes Empirical Bayes (BEB) approach, which assigns a prior to the model parameters and integrates over their uncertainties, to calculate the posterior probabilities of a specific codon site and to identify those most likely to be under positive selection [85], [86]. In addition, the Bonferroni correction [87] was applied for multiple testing in the analysis.

In all PAML analyses, the tree topology ((((Cetartiodactyla, Perissodactyla), Carnivora), Chiroptera), ((Rodentia, Lagomorpha), Primates), Marsupialia) (Figure 1 and Figure 2), based on an analysis of nearly 10,000 bp of 18 genes from 64 mammalian species [88], [89] was used. In fact, to avoid a possible effect of tree topology used on the results, we also conducted the analyses using alternative tree topologies among the published mammalian phylogenies [90], [91], [92], [93] and different tree topologies at family level, and obtained the same results.

## Supporting Information

Figure S1 The mature protein alignment of 59 *leptin* sequences used in this study. The positions of four α-helices (helices A-D) and a distorted helix E in the CD loop are indicated. The species in Cetacea and Pinnipedia are crossed.
(TIF)

Figure S2 The mature protein alignment of 24 *leptin receptor* (*LPR*) sequences used in this study. The positions of extracellular, transmembrane, and intracellular regions, as well as the binding regions for leptin (cytokine receptor homology; CRH2) are indicated. The species in Cetacea and Pinnipedia are crossed.
(TIF)

Figure S3 NJ trees based on *leptin* alignment (501 nt; 59 sequences). MP and Bayesian analyses (AIC model: GTR+G; Base frequencies: A = 0.2405; C = 0.3097; G = 0.2365 and T = 0.2133; Transition/transversion ratio: R[AC] = 0.8717; R[AG] = 4.7477; R[AT] = 0.2437; R[CG] = 0.9188; R[CT] = 3.5498; R [GT] = 1. 0000; gamma shape = 0.9150) produced similar tree topologies to those of NJ analyses with similar nodal supports. The bootstrap supports shown on the nodes are calculated from NJ/MP/Bayesian analyses. Those not shown on the nodes are poorly supported by all the three analyses.
(TIF)

Figure S4 NJ trees based on *LPR* alignment (3519 nt; 24 sequences). MP and Bayesian analyses (AIC model: TVM+G; Base frequencies: A = 0.2723; C = 0.2327; G = 0.2286 and T = 0.2665; Transition/transversion ratio: R[AC] = 1.5455; R[AG] = 5.1963; R[AT] = 0.5967; R[CG] = 1.1684; R[CT] = 5.1963; R [GT] = 1.00 00; gamma shape = 0.8000) produced similar tree topologies to those of NJ analyses with similar nodal supports. The bootstrap supports shown on the nodes are calculated from NJ/MP/Bayesian analyses. Those not shown on the nodes are poorly supported by all the three analyses.
(TIF)

Table S1 List of taxonomic samples and sequences used in this study
(DOC)

Table S2 PAML results for branches a-p except for branch b and m in Figure 1.
(DOC)
