# Supplementary figures and images for: Evidence for Positive Selection on the Leptin Gene in Cetacea and Pinnipedia
Source: PLoS One. 2011 Oct 27;6(10):e26579. doi: 10.1371/journal.pone.0026579 (PMC3203152; doi:10.1371/journal.pone.0026579)

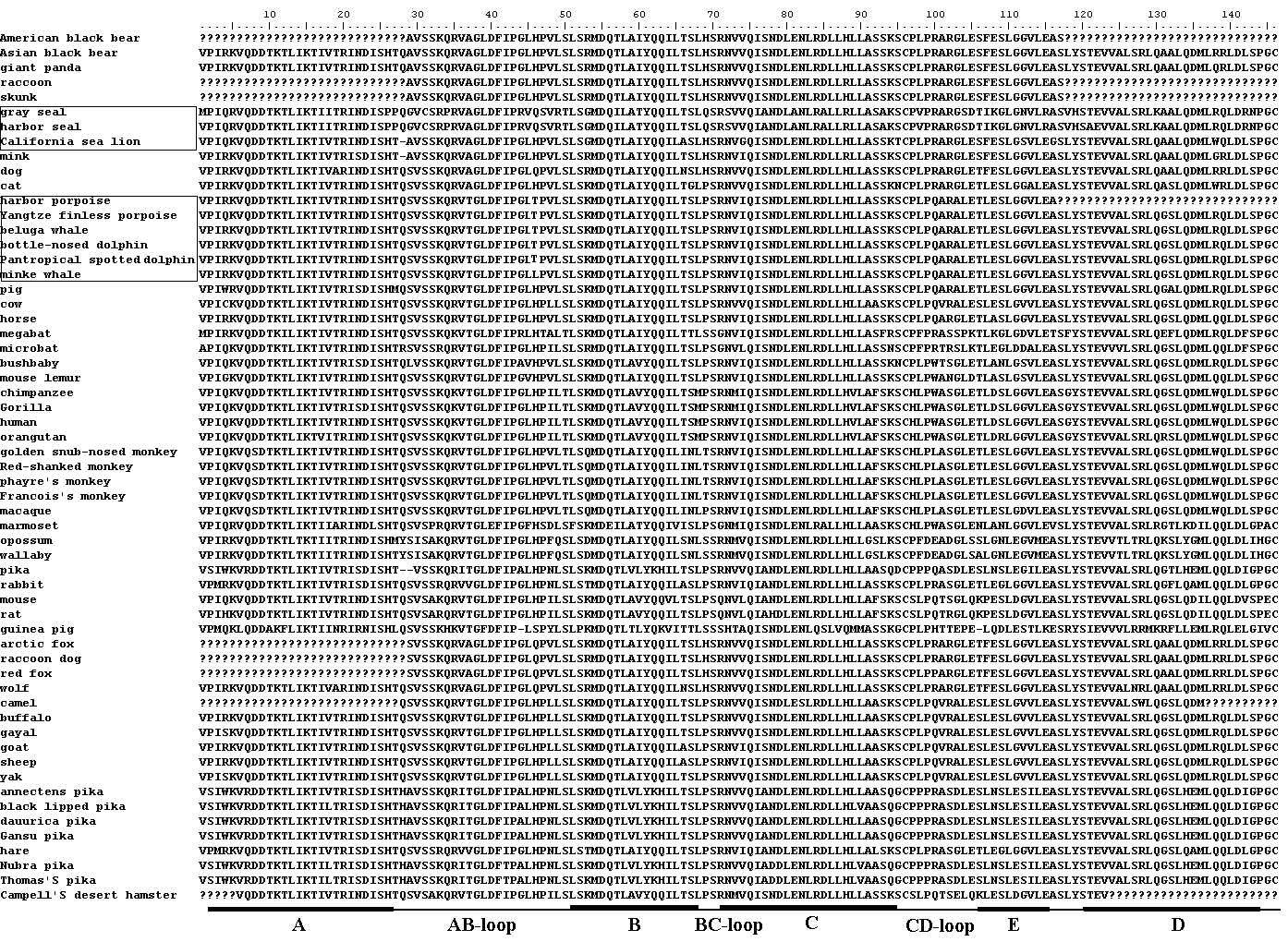

Supplement: Figure S1 — The mature protein alignment of 59 leptin sequences used in this study. The positions of four α-helices (helices A-D) and a distorted helix E in the CD loop are indicated. The species in Cetacea and Pinnipedia are crossed. (TIF) [file pone.0026579.s001.tif]

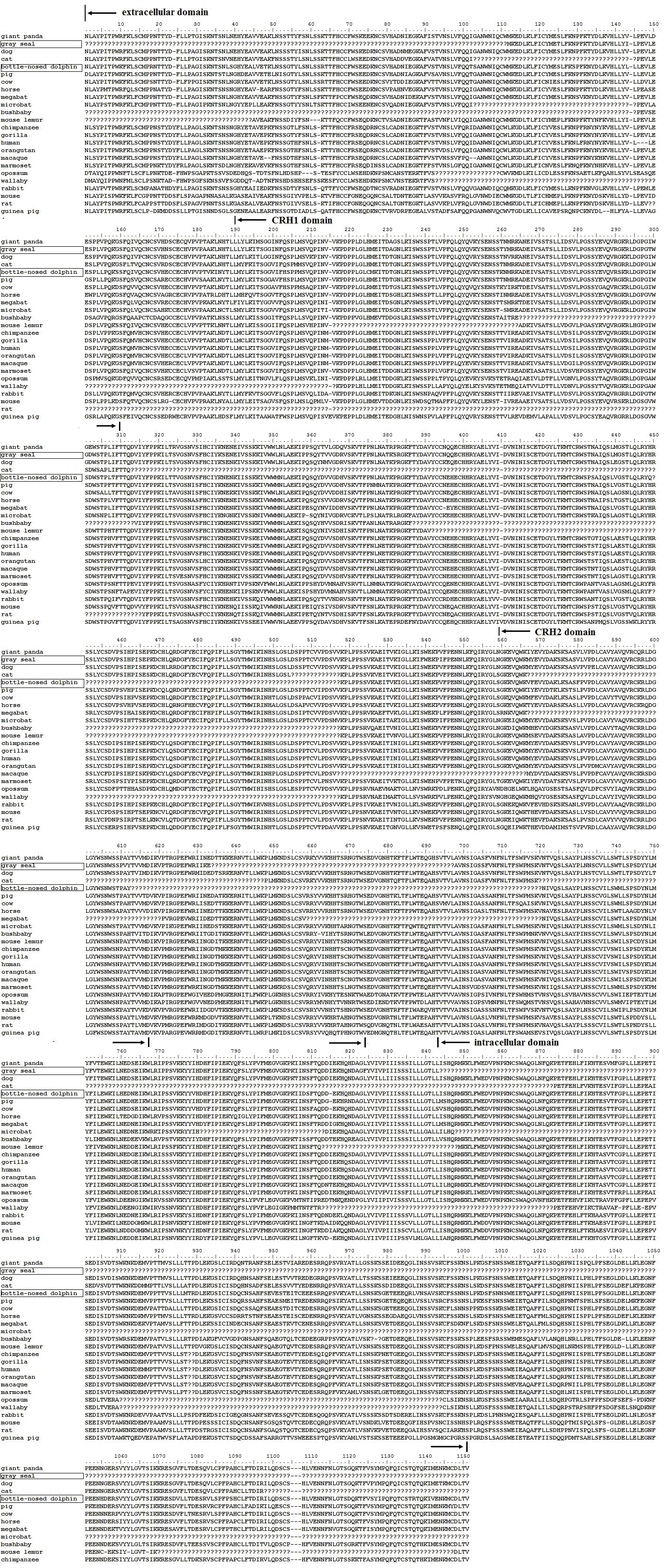

Supplement: Figure S2 — The mature protein alignment of 24 leptin receptor (LPR) sequences used in this study. The positions of extracellular, transmembrane, and intracellular regions, as well as the binding regions for leptin (cytokine receptor homology; CRH2) are indicated. The species in Cetacea and Pinnipedia are crossed. (TIF) [file pone.0026579.s002.tif]

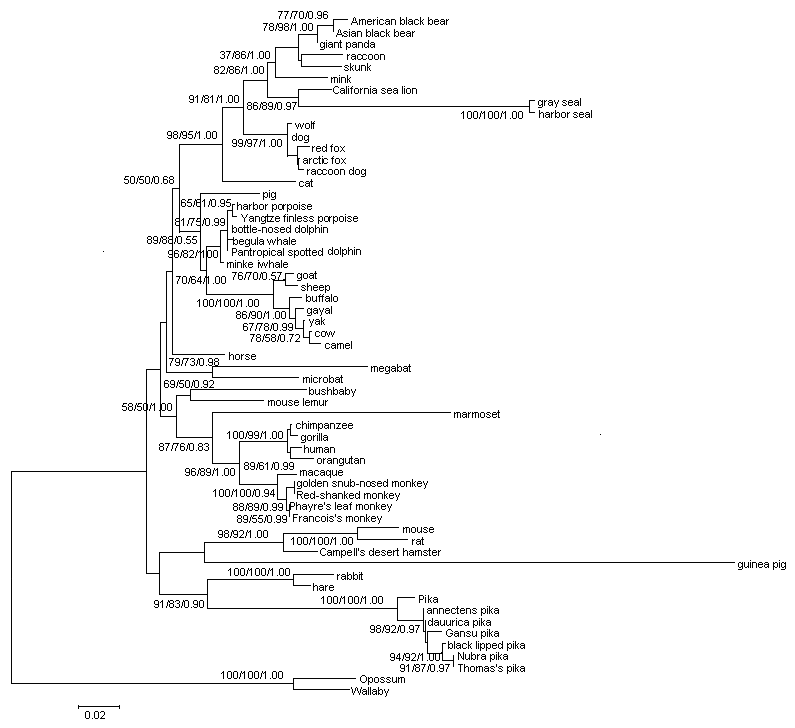

Supplement: Figure S3 — NJ trees based on leptin alignment (501 nt; 59 sequences). MP and Bayesian analyses (AIC model: GTR+G; Base frequencies: A = 0.2405; C = 0.3097; G = 0.2365 and T = 0.2133; Transition/transversion ratio: R[AC] = 0.8717; R[AG] = 4.7477; R[AT] = 0.2437; R[CG] = 0.9188; R[CT] = 3.5498; R [GT] = 1. 0000; gamma shape = 0.9150) produced similar tree topologies to those of NJ analyses with similar nodal supports. The bootstrap supports shown on the nodes are calculated from NJ/MP/Bayesian analyses. Those not shown on the nodes are poorly supported by all the three analyses. (TIF) [file pone.0026579.s003.tif]

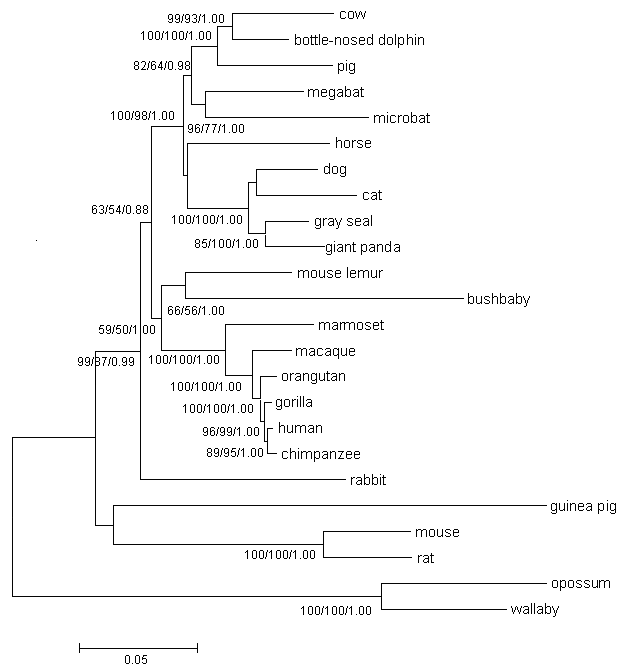

Supplement: Figure S4 — NJ trees based on LPR alignment (3519 nt; 24 sequences). MP and Bayesian analyses (AIC model: TVM+G; Base frequencies: A = 0.2723; C = 0.2327; G = 0.2286 and T = 0.2665; Transition/transversion ratio: R[AC] = 1.5455; R[AG] = 5.1963; R[AT] = 0.5967; R[CG] = 1.1684; R[CT] = 5.1963; R [GT] = 1.00 00; gamma shape = 0.8000) produced similar tree topologies to those of NJ analyses with similar nodal supports. The bootstrap supports shown on the nodes are calculated from NJ/MP/Bayesian analyses. Those not shown on the nodes are poorly supported by all the three analyses. (TIF) [file pone.0026579.s004.tif]
