# Supplementary material for: Evidence for Positive Selection on the Leptin Gene in Cetacea and Pinnipedia
Source: PLoS One. 2011 Oct 27;6(10):e26579. doi: 10.1371/journal.pone.0026579 (PMC3203152; doi:10.1371/journal.pone.0026579)
Supplement: Table S1 — List of taxonomic samples and sequences used in this study (DOC) [file pone.0026579.s005.doc]

**Additional file Table 1**
