# Supplementary material for: Evidence for Positive Selection on the Leptin Gene in Cetacea and Pinnipedia
Source: PLoS One. 2011 Oct 27;6(10):e26579. doi: 10.1371/journal.pone.0026579 (PMC3203152; doi:10.1371/journal.pone.0026579)
Supplement: Table S2 — PAML results for branches a-p except for branch b and m in Figure 1. (DOC) [file pone.0026579.s006.doc]

**Additional file Table 2**
